# Concordance rates among dermatopathologists and Mohs surgeons in frozen section Mohs slides: A systematic review

**DOI:** 10.1016/j.jdin.2023.09.009

**Published:** 2023-10-21

**Authors:** Henry O. Herrera, Marina K. Ibraheim, Conroy Chow, Naomi Lawrence, Jeremy S. Bordeaux, Ashley Elsensohn

**Affiliations:** a Department of Dermatology, Case Western Reserve University, Cleveland, Ohio; b Department of Dermatology, Loma Linda University Health, Loma Linda, California; c Department of Dermatology, Cooper Hospital, Rowan University, Camden, New Jersey; d Department of Dermatology, University Hospitals Cleveland Medical Center, Case Western Reserve University, Cleveland, Ohio; e Department of Pathology, Loma Linda University Health, Loma Linda, California

**Keywords:** concordance, dermatopathology, histopathology, Mohs micrographic surgery

*To the Editor:* Rapid and accurate evaluation of histopathologic specimens is an essential skill that Mohs surgeons employ as part of their regular clinical practice. Both Mohs surgeons and dermatopathologists are trained to interpret frozen section margins, and a recent study demonstrated excellent concordance in the interpretation of Mohs frozen sections between these 2 groups.^1^ However, it is not well established which areas of discordance predominate and what are the reasons for this discordance. Herein, we examined the literature assessing concordance between Mohs surgeons and dermatopathologists for frozen sections taken during the Mohs procedure.

A systematic literature search of PubMed and Embase using the preferred reporting items for systematic reviews and meta-analyses guidelines included the following terms: “Mohs surgery,” “histopathology,” “dermatopathology,” “pathology,” and “concordance” (Fig 1). Articles in which the authors did not assess slide comparisons were excluded. Concordance was determined by agreement on slide diagnosis or tumor presence or absence (Supplementary Table I, available via Mendeley at https://data.mendeley.com/datasets/phshjzznxn/2).

**Fig 1:**
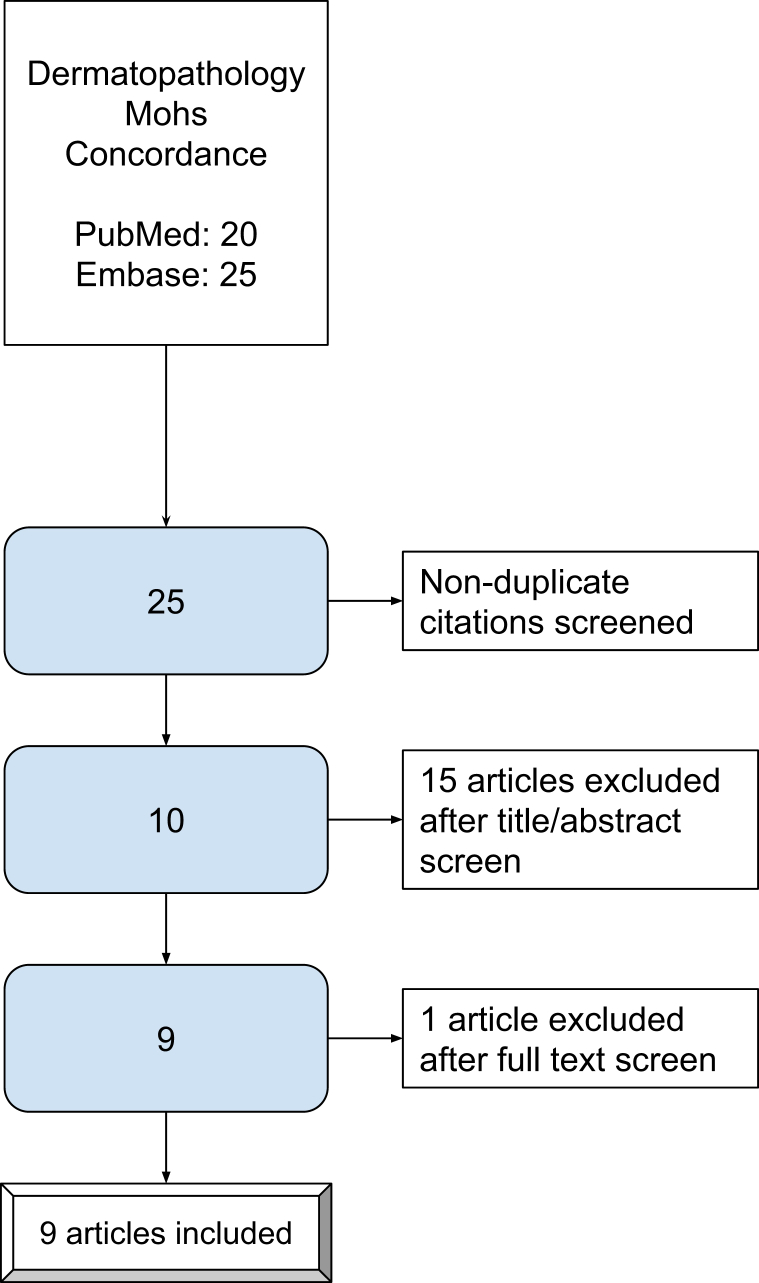
Preferred reporting items for systematic reviews and meta-analyses diagram.

A total of 9 retrospective studies interpreting 11,190 slides were included. The majority of the studies (8/9) were conducted at academic centers, with 19 fellowship-trained Mohs surgeons, and 3 studies included 18 Mohs fellows. One study (1/9) was conducted at a private practice.^2^ The concordance rate ranged from 94.90% to 99.79%, with the highest concordance rate from an academic institution with fellowship-trained Mohs surgeons. Tumors included were basal cell carcinoma (8/9 studies), squamous cell carcinomas (8/9 studies), sebaceous carcinoma (1/9 studies), mucinous carcinoma (1/9 studies), and melanoma (1/9 studies).

In total, 37 discordant slides (Fig 2) were reported (Supplementary Table I, available via Mendeley at https://data.mendeley.com/datasets/phshjzznxn/2). Of these, in 18/37 (48.6%) cases, Mohs micrographic surgery diagnosed a slide as tumor-positive, whereas dermatopathology reported it as tumor-free. Reasons for this discrepancy included the misidentification of a thick section as tumor-positive and the misinterpretation of inflammation as a tumor. In a total of 15/37 (40.5%) discordant cases, Mohs micrographic surgery diagnosed a slide as tumor-free, whereas dermatopathology reported it as tumor-positive. Three discordant slides from 1 patient (3/37, 8.1%) were due to dermatopathology diagnosing melanoma in situ, lentigo maligna type, which was not identified by Mohs micrographic surgery.^2^ One discordant slide (1/37, 2.7%) was from a case in which the Mohs surgeon diagnosed the slide a squamous cell carcinoma in situ, whereas the dermatopathologist diagnosed it as a superficial basal cell carcinoma.

**Fig 2:**
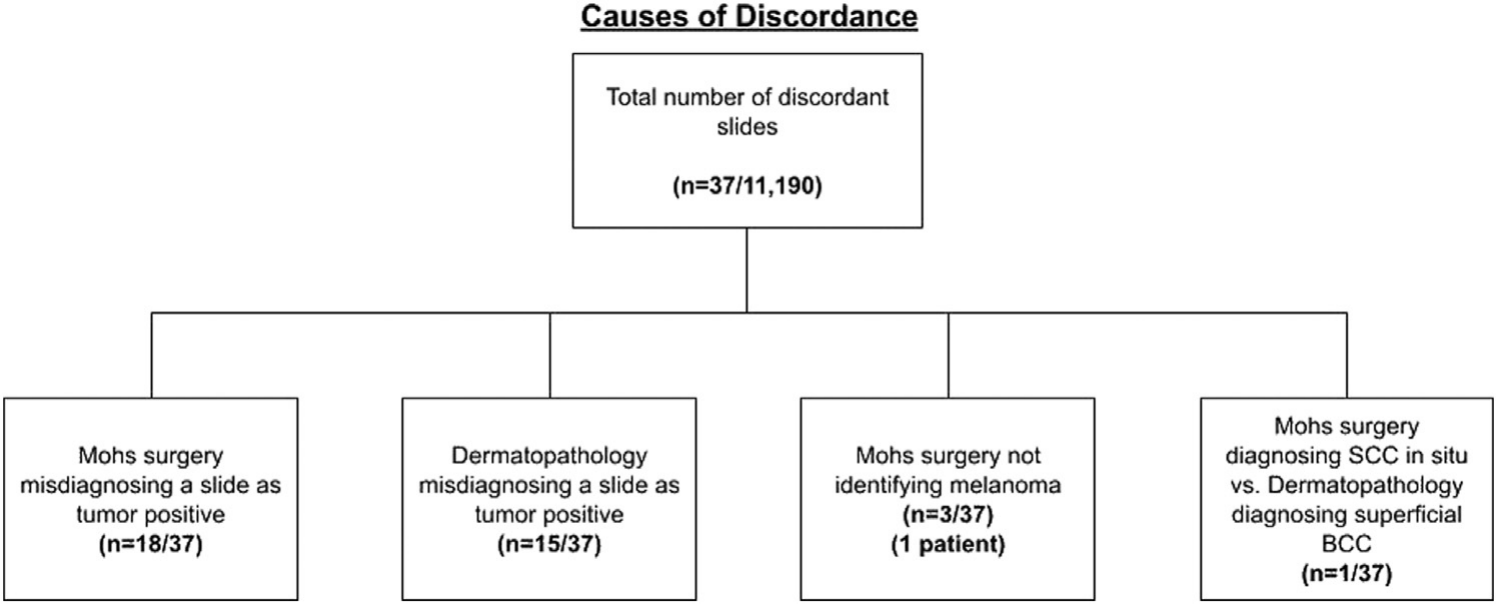
Discordance between Mohs micrographic surgery and dermatopathology.

Overall, this systematic literature review reveals a high concordance rate between Mohs surgeons and dermatopathologists, particularly among fellowship-trained Mohs surgeons at academic institutions. Limitations of this study include a small sample size, the retrospective nature of the study, and the predominance of studies at academic centers with fellowship-trained Mohs surgeons or fellows. Future studies are necessary to evaluate concordance in other practice models and should involve non–fellowship-trained Mohs surgeons. Given the increase in use of Mohs micrographic surgery for melanoma, additional studies are needed to evaluate its accuracy in the diagnosis of melanoma and rare cutaneous tumors.^3^ Finally, identifying measures that can be implemented in the education of dermatopathologists and Mohs surgeons to enhance accuracy in the interpretation of frozen histology will be essential to improve concordance and patient outcomes.

## Conflicts of interest

None disclosed.
